# Analytic continuation and incomplete data tomography

**DOI:** 10.14312/2399-8172.2021-2

**Published:** 2021-03-04

**Authors:** Gengsheng L. Zeng, Ya Li

**Affiliations:** 1 Department of Computer Science, Utah Valley University, Orem, USA; 2 Department of Radiology and Imaging Sciences, University of Utah, Salt Lake City, USA; 3 Department of Mathematics, Utah Valley University, Orem, USA

**Keywords:** analytic continuation, medical imaging, incomplete data tomography

## Abstract

A unique feature of medical imaging is that the object to be imaged has a compact support. In mathematics, the Fourier transform of a function that has a compact support is an entire function. In theory, an entire function can be uniquely determined by its values in a small region, using, for example, power series expansions. Power series expansions require evaluation of all orders of derivatives of a function, which is an impossible task if the function is discretely sampled. In this paper, we propose an alternative method to perform analytic continuation of an entire function, by using the Nyquist–Shannon sampling theorem. The proposed method involves solving a system of linear equations and does not require evaluation of derivatives of the function. Noiseless data computer simulations are presented. Analytic continuation turns out to be extremely ill-conditioned.

## 1 Introduction

It is known that for stable image reconstruction using noisy data, measurements must be sufficiently acquired. There are many data sufficiency conditions that are proposed. For example, in cone-beam imaging, Tuy’s condition must be satisfied [1]. Tuy’s condition requires that the cone focal point trajectory must interest every plane that cuts the object being imaged. In PET (positron emission tomography), Orlov’s condition must be satisfied [2]. Orlov’s condition requires that every great circle must intersects the trajectory of the unit vector that is the direction of the projection rays. In MRI (magnetic resonance imaging), the k-space (i.e., the Fourier space) preferably should be fully sampled.

As theoretical curiosity, one would wonder whether it is possible to reconstruct the image using incomplete data. This subject has been systematically discussed in Natterer’s book [3], where the incomplete data situations are classified in to 3 categories: limited angle problems, exterior problems, and truncated problems. For the limited angle problems and exterior problems, the inversion is so seriously ill-posed that it is hopeless to have any practical value.

The goal of this paper is not to develop an algorithm that can be used immediately in practice. The motivation of this paper is purely theoretical. Assuming that we live in an ideal world without any noise around, we investigate whether it is possible to reconstruct an image using incomplete measurements.

In medical image, different modalities have their unique ways to acquire data. MRI is the most relevant modality to this paper, because MRI measurements are in the Fourier domain. In PET or CT, the measurements are in the spatial-domain. If the projection measurements are truncated, their Fourier transform does not exist. When no point in the Fourier domain is measured, this situation is not relevant to this paper. Under certain conditions, truncated projections may produce satisfactory images, even though Fourier measurements are not available. If the projections are not truncated, the Fourier transform can be taken, and the central slice theorem applies. The central slice theorem indicates what frequency components are measured and what frequency components are not measured.

When Fourier measurements are incomplete, we can try to reconstruct the image using the available measurements and some prior constraints. As implied in this paper, trying to estimate the unmeasured data may not be fruitful.

## 2 Methods

### 2.1 Mathematical foundation

A function has a compact support if it is zero outside of a compact set that is closed and bounded. The Fourier transform of a compactly supported function is an entire function. An entire function is a complex-valued function that is holomorphic at all finite points over the whole complex plane. A holomorphic function is complex differentiable at every point of its domain. Any holomorphic function is infinitely differentiable and equal, locally, to its own Taylor series. A holomorphic function whose domain is the whole complex plane is called an entire function [4].

The possibility of imaging with incomplete data is established as follows, using a one-dimensional (1D) example. The object *f*(*x*) is compactly supported, for example, defined on [−½, ½] and *f*(*x*) = 0 elsewhere. Let *F*(*ω*) be the Fourier transform of *f*(*x*). Assume that *f*(*x*) is unknown, *F*(*ω*) is partially known. Without loss of generality, *F*(*ω*) is assumed to be known in a small region around the point *ω* = 0. One can evaluate derivatives of F(*ω*) at all orders, and thus construct the Taylor series of *F*(*ω*) at *ω* = 0. Since *F*(*ω*) is an entire function, this Taylor series converges to *F*(*ω*) in the entire complex plane. In other words, *F*(*ω*) becomes known in the entire complex plane through analytic continuation.

This analytic continuation of *F*(*ω*) is actually of no use in practice, because most real world measurements are discrete. This fact inhibits the evaluation of derivatives of *F*(*ω*), and thus the Taylor series of *F*(*ω*) cannot be obtained.

### 2.2. Lagrange interpolation method

Other than the Taylor series expansion, the Lagrange interpolation formula can be an alternative method to perform analytic continuation [5]. The essential idea of the Lagrange interpolation formula is to find the lowest order polynomial that passes through given points.

We do not believe that the Fourier transform of a compactly supported function in medical imaging behaves like a polynomial. The energy of a compactly supported function *f*(*x*) is finite. Parseval’s Theorem tells us that the energy of its Fourier transform *F*(*ω*) is the same and finite. As |*ω*|➔∞, we must have *F*(*ω*) ➔ 0. Hence, *F*(*ω*) cannot behave as a polynomial, because the magnitude of a polynomial tends to infinity as the magnitude of the variable tends to infinity.

### 2.3 Nyquist-Shannon method

If a spatial-domain function is band-limited, then this signal can be represented by its discrete samples, and the sampling interval is inversely proportional to the bandwidth. If we switch the roles of these two domains, the spatial-domain function *f*(*x*) is spatially bounded and the corresponding Fourier domain function *F*(*ω*) can be represented by its discrete samples. The Fourier domain sampling interval Δ*ω* is inversely proportional to the spatial-domain object size. According to the Nyquist-Shannon Theorem, we can express the complex Fourier domain function *F*(*ω*) by its own samples *F*(*n*Δ*ω*) as

(1) $$F(\omega )=\sum_{n=-\infty }^{\infty }F(n\Delta \omega )\times \mathrm{sinc}\left(\frac{\omega -n\Delta \omega }{\Delta \omega }\right)$$

where the sinc function is defined as

(2) $$sinc(x)=\{\begin{array}{c} \frac{\sin(\pi x)}{\pi x}\;\mathrm{if}\;x\neq 0\\ 1\;\mathrm{if}\;x=0 \end{array}$$

Formula (1) is referred to as the Whittaker-Shannon interpolation formula. Formula (1) implies that the function *F*(*ω*) is sufficiently determined by its discrete values *F*(*n*Δ*ω*), where *n* ∈ Z (integers). Because *f*(*x*) is the inverse Fourier transform of *F*(*ω*), the spatial-domain compactly supported function *f*(*x*), in turn, is determined by the samples *F*(*n*Δ*ω*).

According to the fact that *F*(*ω*) ➔ 0 as |*ω*| ➔ ∞, we can obtain an approximate expression of (1) by using only a finite number of terms in the summation:

(3) $$F(\omega )\approx \sum_{n=-N}^{N}F(n\Delta \omega )\times \mathrm{sinc}\left(\frac{\omega -n\Delta \omega }{\Delta \omega }\right)$$

It is reasonable to further assume that the function *f*(*x*) is real and *F =F*_*r*_ *+ iF*_*i*_, and then the real part *F*_*r*_(*ω*) is even and the imaginary part *F*_*i*_(*ω*) is odd. Therefore, (3) can be written as

(4a) $$F_{r}(\omega )\approx F_{r}(0)+2\sum_{n=1}^{N}F_{r}(n\Delta \omega )\times \left[\mathrm{sinc}\left(\frac{\omega -n\Delta \omega }{\Delta \omega }\right)+\mathrm{sinc}\left(\frac{\omega +n\Delta \omega }{\Delta \omega }\right)\right]$$

(4b) $$F_{i}(\omega )\approx 2\sum_{n=1}^{N}F_{i}(n\Delta \omega )\times \left[\mathrm{sinc}\left(\frac{\omega -n\Delta \omega }{\Delta \omega }\right)-\mathrm{sinc}\left(\frac{\omega +n\Delta \omega }{\Delta \omega }\right)\right]$$

### 2.4 Proposed method

This part presents the main result of this current paper. We consider a Fourier transform pair: *f*(*x*) and *F*(*ω*), where *f*(*x*) is real and has a compact support, and *F*(*ω*) is complex and entire. The spatial-domain function *f*(*x*) is unknown. The Fourier domain function *F*(*ω*) is measured at discrete points, *ω*_*k*_, *k* = 1, 2, …, *M*, which are in a smaller interval than [−*N, N*]. Let us form 2 real column vectors:

(5) $$p=\left[F_{r}\left(\omega_{1}\right),F_{r}\left(\omega_{2}\right),\ldots ,F_{r}\left(\omega_{M}\right),F_{i}\left(\omega_{1}\right),F_{i}\left(\omega_{2}\right),\ldots ,F_{i}\left(\omega_{M}\right)\right]$$

and

(6) $$u={\left[F_{r}(0),F_{r}(\Delta \omega ),\ldots ,F_{r}(N\Delta \omega ),F_{i}(\Delta \omega ),F_{i}(N\Delta \omega )\right]}^{T}$$

The vector *p* contains the measurements, and the vector *u* contains the unknowns. It is allowed that some of the unknowns are the measurements. The approximations (4a) and (4b) can be written in the matrix form as

(7) Au≈p

where the real (2M) × (2N + 1) matrix *A* is determined according to (4a) and (4b). A numeric algorithm is required to solve the unknown vector *u* from (7). Once the vector *u* is obtained, the spatial-domain function *f*(*x*) is constructed by *u* as follows.

If we consider the compactly supported function *f*(*x*) as one period of a periodic function, then *f*(*x*) has a Fourier series expansion

(8) $$f(x)=\sum_{n=-\infty }^{\infty }c_{n}e^{\frac{i2\pi nx}{T}}\approx \sum_{n=-N}^{N}c_{n}e^{\frac{i2\pi nx}{T}}$$

with Fourier coefficients

(9) $$c_{n}=\frac{1}{T}\int_{-T/2}^{T/2}f(x)e^{\frac{-i2\pi nx}{T}}dx$$

where *T* is the period and can be same as (or larger than) the span of *f*(*x*). Since the Fourier transform of *f*(*x*) is defined as

(10) $$F(\omega )=\int_{-T/2}^{T/2}f(x)e^{-i2\pi \omega x}dx$$

we have

(11) $$c_{n}=\frac{1}{T}F\left(\frac{n}{T}\right)$$

If we choose Δ*ω*=1/T, the Fourier series coefficients can be obtained by solving (7). When the Fourier series (8) is truncated, the summation can be implemented by the (2*N*+2)-point inverse discrete Fourier transform (IDFT) or the inverse fast Fourier transform (IFFT).

(12) $$\sum_{n=0}^{2N+1}c_{n}e^{\frac{i2\pi nx}{2N+2}}$$

where c_N+1_ = 0 and c_N+1+k_ = c*_N+1-k_, *k* = 1, … , *N*.

Solving for *u* from (7) is challenging, because the system is seriously ill-posed. In the computer simulations in this paper, no noise is added to the measurements *p*. The computer round-off errors are already serious enough to make the solution deviate from the true solution. The following approaches can be used to find the vector *u*.

#### Approach 1:

The Moore-Penrose pseudo inverse with a tolerance *tol*. This approach finds the singular value decomposition (SVD) of the matrix *A* and replaces the singular values that are smaller than *tol* by zeros before calculating the generalized inverse of *A*.

(13) $$u=A^{†}p$$

#### Approach 2:

(14) $$\underset{u}{\min}\left(\|Au-p{\|}_{2}+\alpha \|u{\|}_{2}\right)$$

#### Approach 3:

(15) $$\underset{u}{\min}\left(\|Au-p{\|}_{1}+\alpha \|u{\|}_{2}\right)$$

#### Approach 4:

(16) $$\underset{u}{\min}\left(\|Au-p{\|}_{\infty }+\alpha \|u{\|}_{2}\right)$$

#### Approach 5:

(17) $$\underset{u}{\min}\left(\|u{\|}_{2}\right)$$

Subject to

(18) Au=p

#### Approach 6:

(19) $$\underset{u}{\min}\left(\|u{\|}_{1}\right)$$

Subject to (18).

#### Approach 7:

(20) $$\underset{u}{\min}\left(\|u{\|}_{\infty }\right)$$

Subject to (18).

### 2.5 Applications to medical imaging

**Figure 1:**
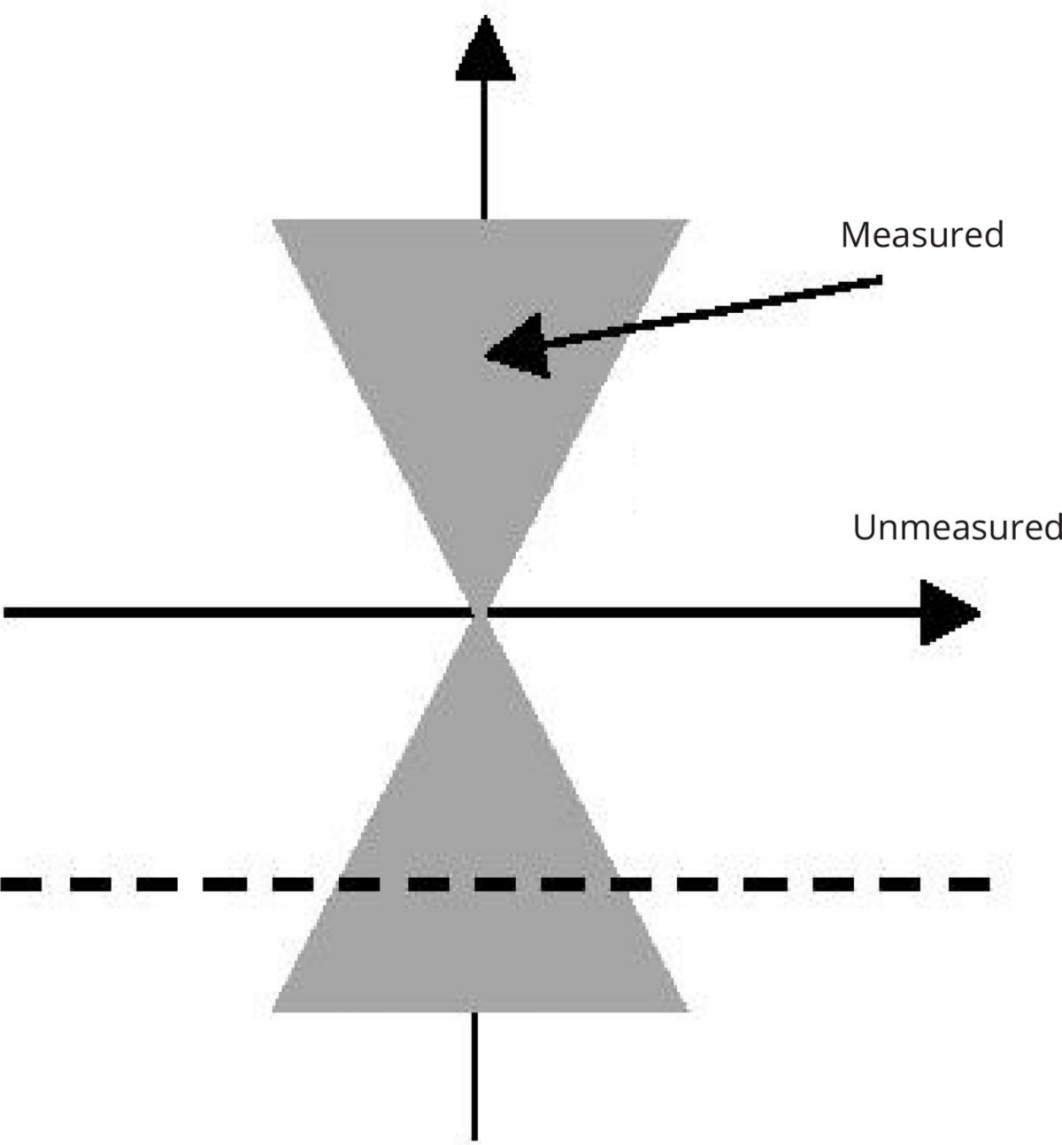
Illustration of a 2D Fourier space when the angular sampling is less than 180o. The shaded regions are measured, while the unshaded regions are not measured. In theory, one can use analytic continuation to estimate the data in the unmeasured regions.

One application of the proposed method is in limited angle tomography, where the Radon transform is only available in an angular range smaller than 180^o^. According to the central slice theorem, in the two-dimensional (2D) Fourier domain, two angular sections are measured, and two remaining angular sections are not, as illustrated in Figure 1.

The measured Fourier components are in the shaded regions. In theory, it is possible to complete the unmeasured Fourier components by line-by-line (which can be row-by-row, or column-by-column) analytic continuation. One analytic continuation method is suggested in Section 2.4.

Another application of the proposed method is in fast MRI, where the k-space is not completely measured. The unmeasured k-space data can be estimated from measured data. If this analytic continuation technology works, MRI procedures can be sped up significantly.

## 3 Results

The first computer simulation considered a 1D function *f*(*x*) that was composed of two boxcars. The Fourier transform of a boxcar function is a sinc function. Therefore, the closed-form of *F*(*ω*) in this case was known. It was assumed that 64 uniform discrete samples of *F*(*ω*) were sufficient to represent the function.

We measured the first 16 frequency components and measured additional 135 components within the measured range. The condition number of *A*^*T*^*A* was 6.0325×10^17^. The strategy of the proposed method is to over-sample the region where data is available. However, when we used additional 1350 components (instead of 135 components) within the measured range, the condition number of *A*^*T*^*A* worsened to 3.4168×10^18^.

The following parameters were used for this simulation: Approach 1: *tol* = 10^−14^. Approach 2: α = 10^−9^. Approach 3: α = 10^−8^. Approach 4: α = 0.

The second simulation was with a Shepp-Logan phantom, for which we did not have a closed-form expression for its Fourier transform. To work around this problem, we first used a computer simulated digitized Shepp-Logan phantom in a 256×256 array that was column-by-column zero-padded so that each column had 2560 pixels. After taking 2560-point 1D DFT, we obtained an over-sampled Fourier spectrum. Among these 2560 samples, we chose the first 100 samples as our measurements. These low frequency 100 components were used to estimate the unmeasured frequency components using the method proposed in Section 2.4.

The DFT assumes discrete and periodic *f*(*x*), as well as discrete and periodic *F*(*ω*). The actual *F*(*ω*) is aperiodic, because the actual *f*(*x*) is continuous. The errors introduced by discretization of *f*(*x*) can be reduced by using smaller sampling intervals. For example, using an array size of 1024×1024 or 2048×2048 to represent the Shepp-Logan phantom.

**Figure 2:**
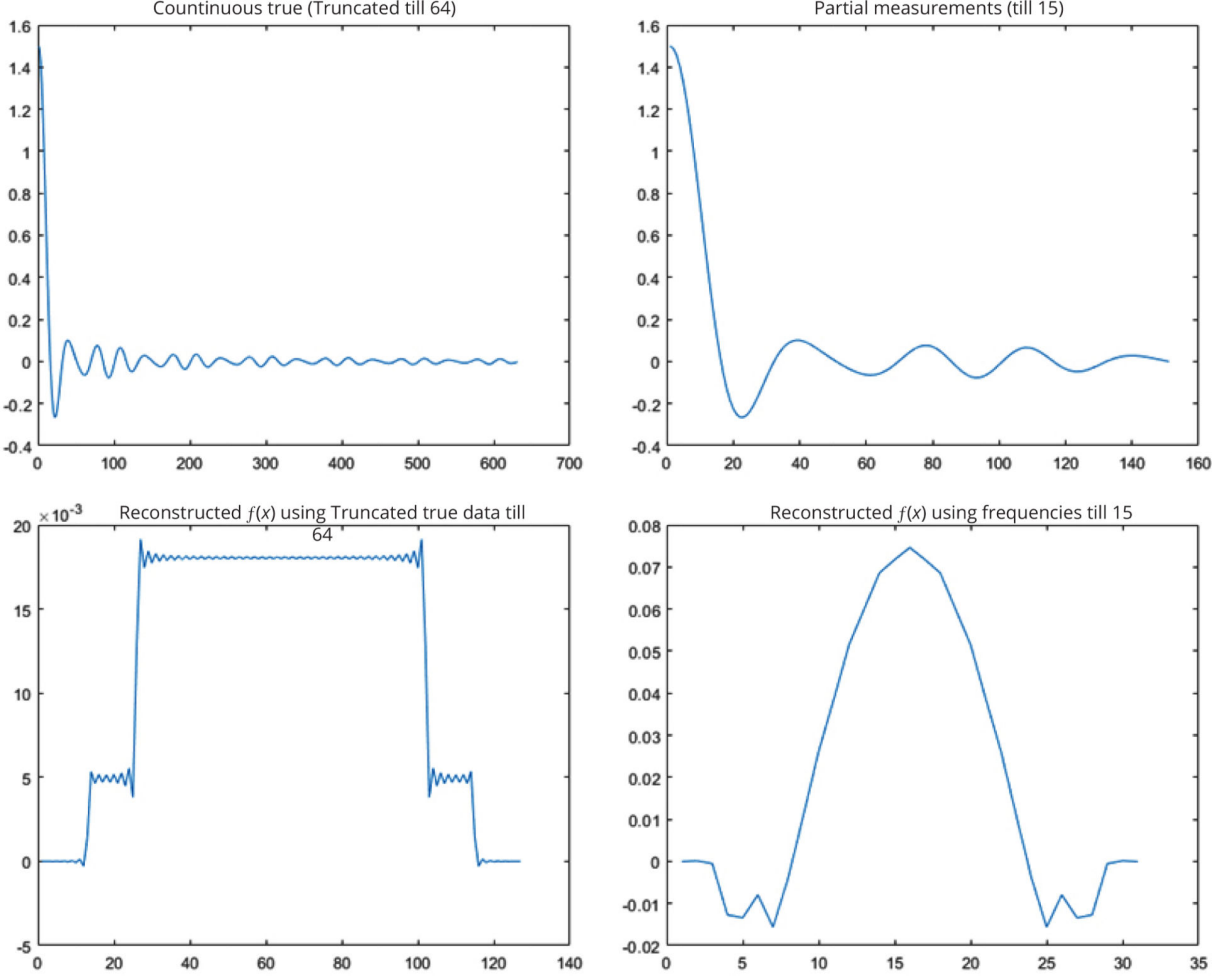
Fourier domain signal *F*(*ω*) (in the upper row) and its reconstruction *f*(*x*) (in the lower row), up to 64 samples and up to 15 samples, respectively.

**Figure 3:**
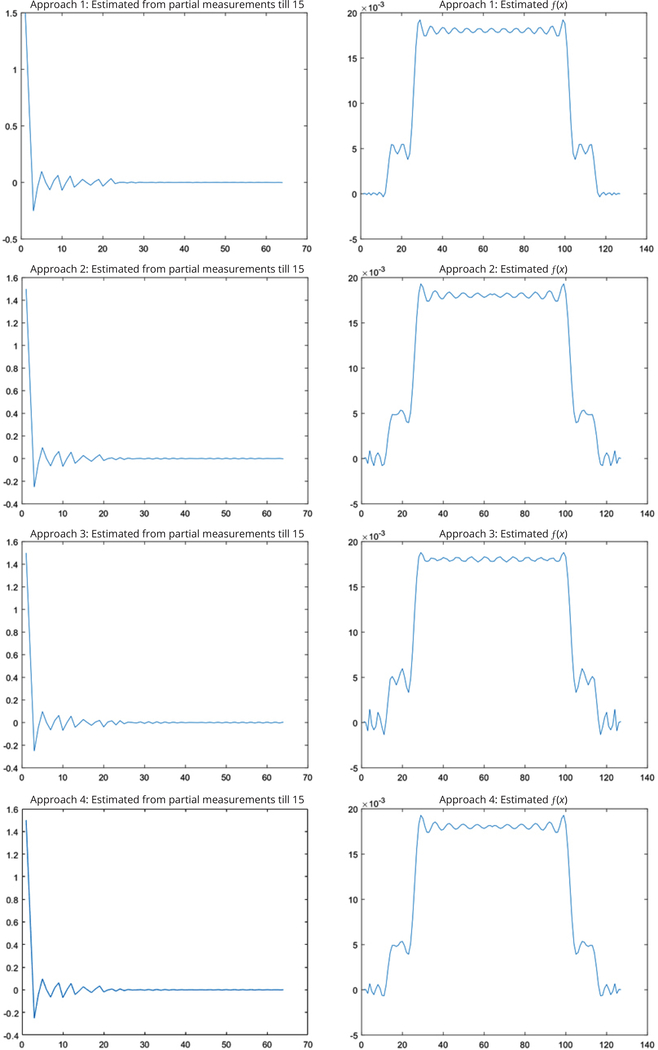

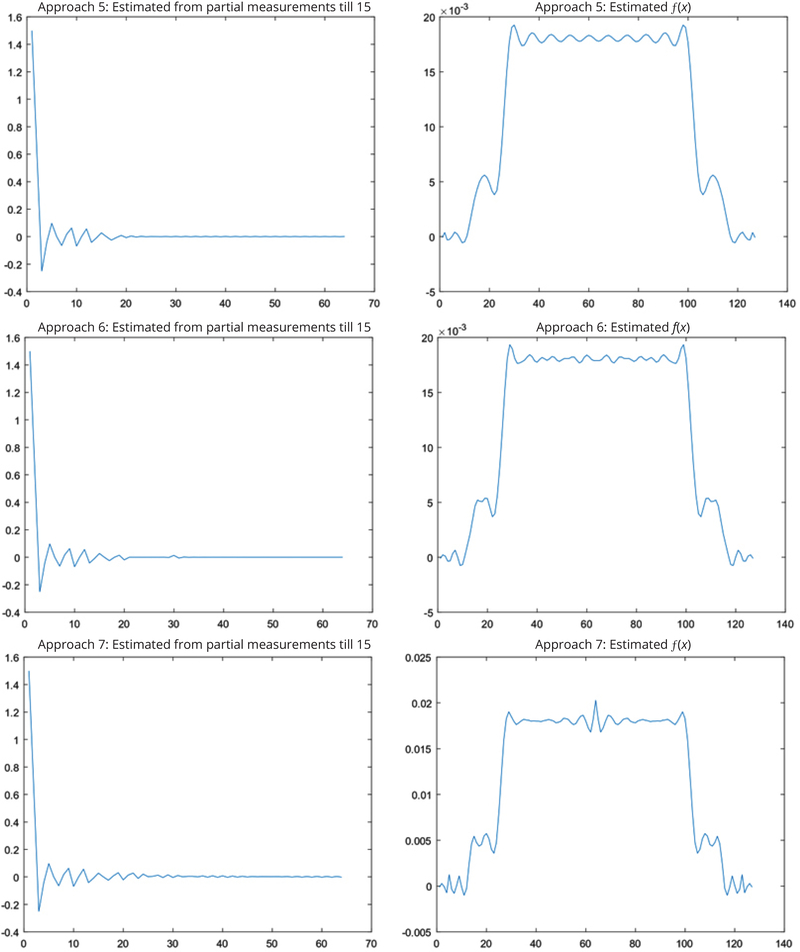
Results for the first simulation. Estimated Fourier components *F*(*ω*) and their reconstructions *f*(*x*) using the proposed 7 approaches.

**Figure 4:**
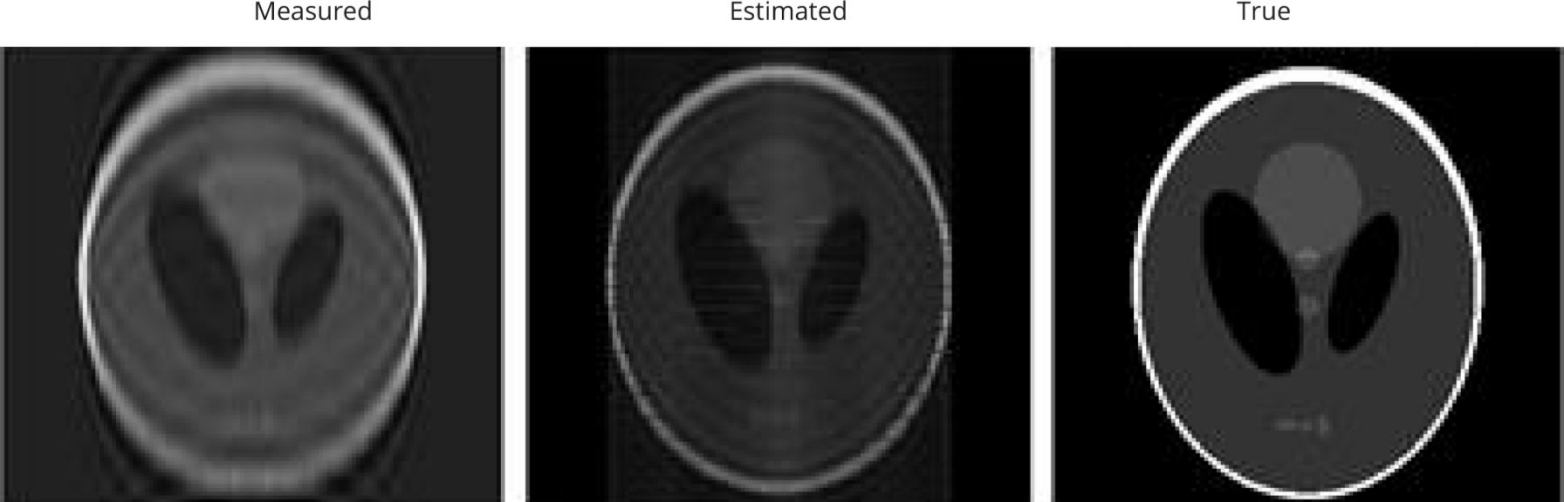
(a) Reconstructed result from measured data, (b) Reconstructed result for the second simulation, using approach #1, (c) The true phantom.

Figure 2 shows the Fourier domain signals and their associated inverse DFT reconstructions. All computer simulation results for the simulations are shown in Figures 3 and 4.

In the first simulation, it was assumed that 64 samples were good enough to represent the original signal. Frequency components were available only lower than sample #15. Table 1 lists the mean-squared-error between the spatial-domain curve (on the right of Figure 3) from the estimated Fourier components and the spatial-domain curve (at the lower left of Figure 2) from the measured Fourier components, using all 7 estimation methods.

In the second simulation, frequency components lower than #10 were available. Three images are shown in Figure 4. The left figure is the reconstructed image from measured data, where the frequency components are in the very low frequency range. The middle figure is the reconstructed image for the estimated data, using approach #1. Some higher frequency components are recovered by the data extension algorithm. The right figure is the true phantom. The mean-squared-error between the Left image and the Right image is 1413, while the mean-squared-error between the Left image and the Right image is 847. The error reduction is approximately 40%.

## 4 Discussion

This paper concerns about data completeness in medical imaging. Since the patient body has a finite support, its Fourier transform is holomorphic. A holomorphic function can be uniquely determined by its values in a small region.

In MRI, this theory suggests that if the k-space is continuously ‘sampled’ without any noise in a very small region, for example, close to the center of the k-space, then the entire k-space can be uniquely estimated.

In CT, this theory suggests that if the projection data is continuously ‘sampled’ without any noise when the x-ray tube continuously rotates in a very small arc, then the entire 360° projections can be uniquely estimated.

Analytic continuation is a powerful tool in mathematics to determine the values of an entire function in a wider region. This paper has developed an analytic continuation method by over-sampling the ‘known’ region and solving a system of linear equations. The system turns out to be seriously ill-posed. Our computer simulations cannot obtain exact estimation even though no noise is added to the measurements. The computer rounding errors are already too large to handle. Seven approaches have been tested. It is interesting to notice that Approach #4 with the infinity norm allows α = 0, while Approaches #1-#3 with L1 or L2 norms require some regularization. The L1 norm forgives outliers, the L2 norm manages the error energy, and the L∞ norm controls the maximum error.

Our results do not imply that the analytic continuation is useless in the real world. Our simulations used sampled data. The analytic continuation requires ‘continuous’ data, which is difficult to implement with today’s computers. It is still an open problem whether analytic continuation is helpful if ‘continuous’ and ‘rounding error free’ computers are available. We believe that denoising must be performed prior to analytic continuation.

## Conclusion

In theory, the Fourier domain data are smooth and uniquely determined by measurements in a small Fourier region. Unfortunately, this theory has not shown any impact in practice due to the ill-condition of its nature. We do not have effective constraints in the Fourier domain to regularize the estimation problem. On the other hand, some constraints are effective in the image domain such as total variation constraint and minimum energy constraint. As a result, the image domain estimation may seem more effective.

## Figures and Tables

**Table 1:** Analysis of mean squared errors for the methods in Figure 3.

Approach index	Mean squared error
1	6.9685 × 10^−5^
2	9.1850 × 10^−5^
3	9.3186 × 10^−5^
4	8.7488 × 10^−5^
5	1.1212 × 10^−4^
6	1.0765 × 10^−4^
7	9.6667 × 10^−5^
